# Supplementary material for: Prioritized mass spectrometry increases the depth, sensitivity and data completeness of single-cell proteomics
Source: Nat Methods. Author manuscript; Available in PMC 2023 May 16. (PMC10172113; doi:10.1038/s41592-023-01830-1)
Supplement: Supplementary Information Supplementary Figs. 1–4 and Tables 1–14. [file NIHMS1890464-supplement-Supplementary_Information_Supplementary_Figs__1_4_and_Tables_1_14_.pdf]

nature methods

Article

https://doi.org/10.1038/s41592-023-01830-1

Prioritized mass spectrometry increases the depth, sensitivity and data completeness of single-cell proteomics

In the format provided by the authors and unedited

Supplementary Information

Prioritized mass spectrometry increases the depth, sensitivity, and data completeness of single-cell proteomics

R Gray Huffman,[1] Andrew Leduc,[1] Christoph Wichmann,[2] Marco di Gioia,[3] Francesco Borriello,[3] Harrison Specht,[1] Jason Derks,[1] Saad Khan,[1] Luke Khoury,[1] Edward Emmott,[1,4] Aleksandra A. Petelski,[1] David H Perlman,[5] Jürgen Cox,[2] Ivan Zanoni,[3] and Nikolai Slavov[1,✉]

[1]Departments of Bioengineering, Biology, Chemistry and Chemical Biology, Single Cell Center, and Barnett Institute, Northeastern University, Boston, MA 02115, USA
[2]Computational Systems Biochemistry Research Group, Max Planck Institute of Biochemistry, Germany
[3]Boston Children's Hospital and Harvard Medical School, Boston, MA 02115, USA
[4]Centre for Proteome Research, Department of Biochemistry & Systems Biology, University of Liverpool, UK
[5]Merck Exploratory Sciences Center, Merck Sharp & Dohme Corp., 320 Bent St. Cambridge, MA 02141, USA
✉ Correspondence: nslavov@northeastern.edu

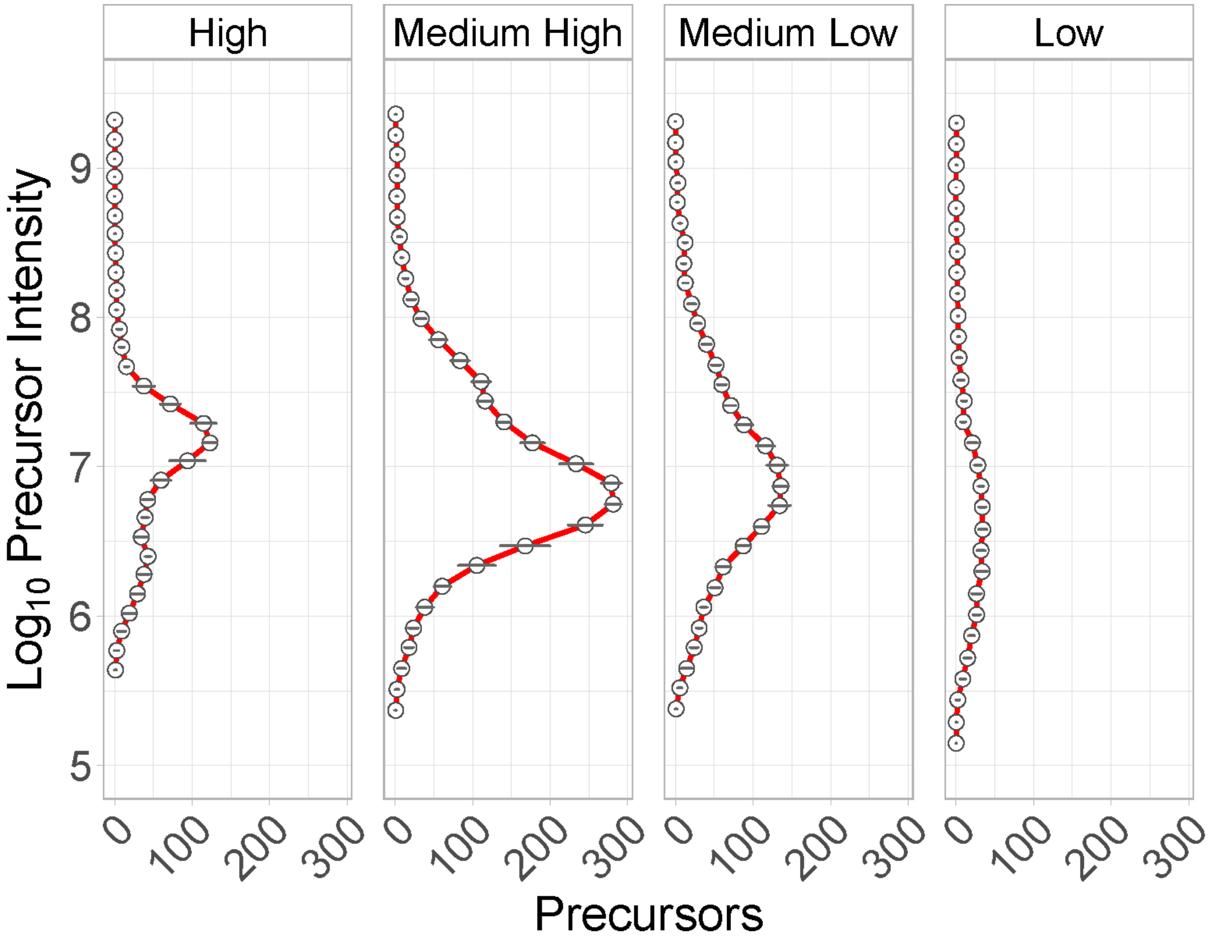

Supplemental Fig. S1 | Precursor intensity comparison for prioritized analyses Comparison of precursor intensities stratified by priority level for the experiments shown in Fig. 2b. n = 8 experiments conducted using prioritized data acquisition. Data are represented by the median, and error bars denote the standard deviation.

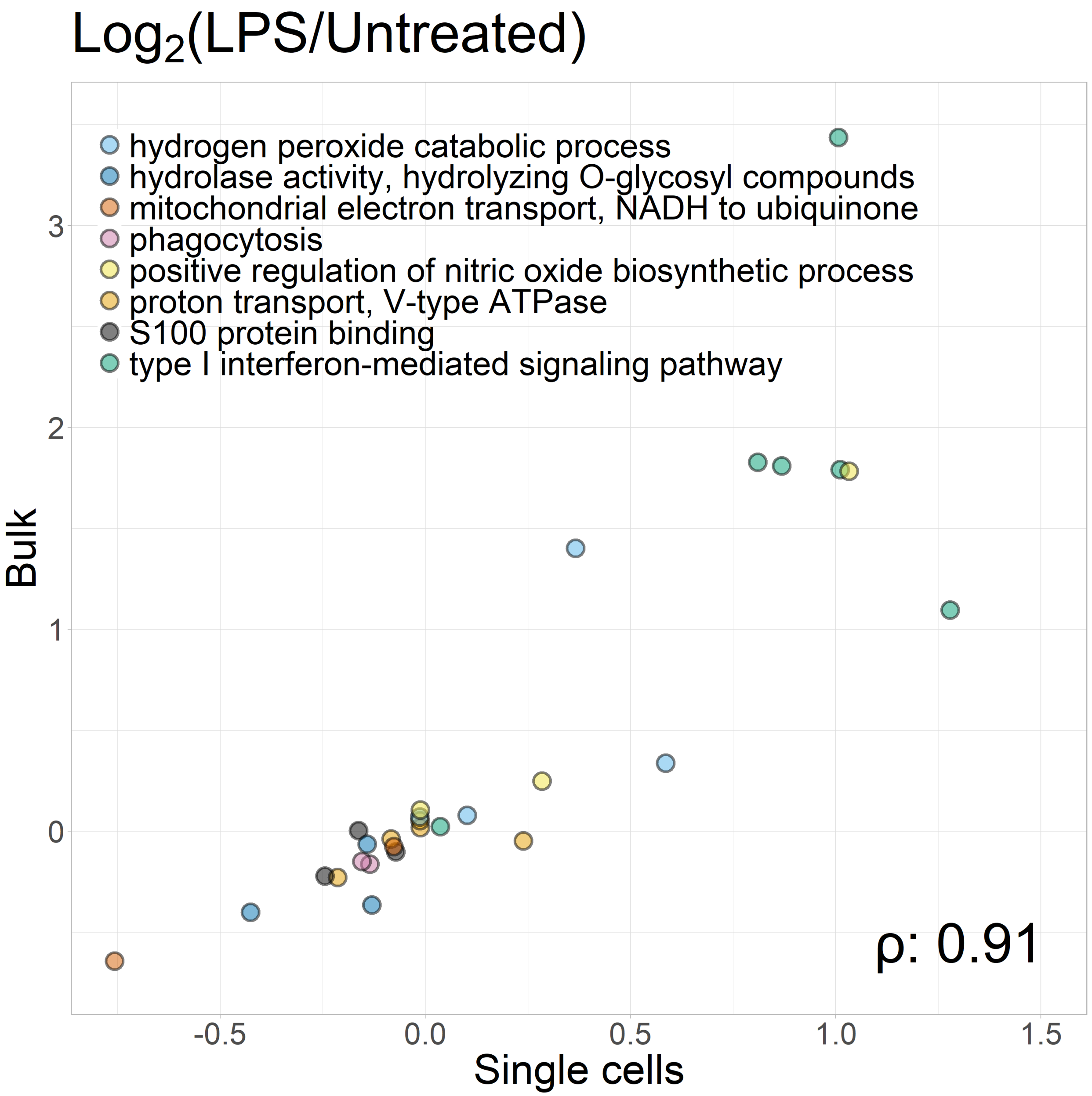

Supplemental Fig. S2 | **GO-term-associated protein fold changes** The protein fold-changes (LPS-treated / untreated macrophages) were estimated both from the single cells and from bulk samples. The corresponding estimates correlate positively with a Spearman correlation $\rho = 0.91$ computed using all 28 proteins shown (p-value $= 2 \times 10^{-11}$). Proteins previously annotated as "other GO terms" in Fig. 4c are explicitly annotated, here.

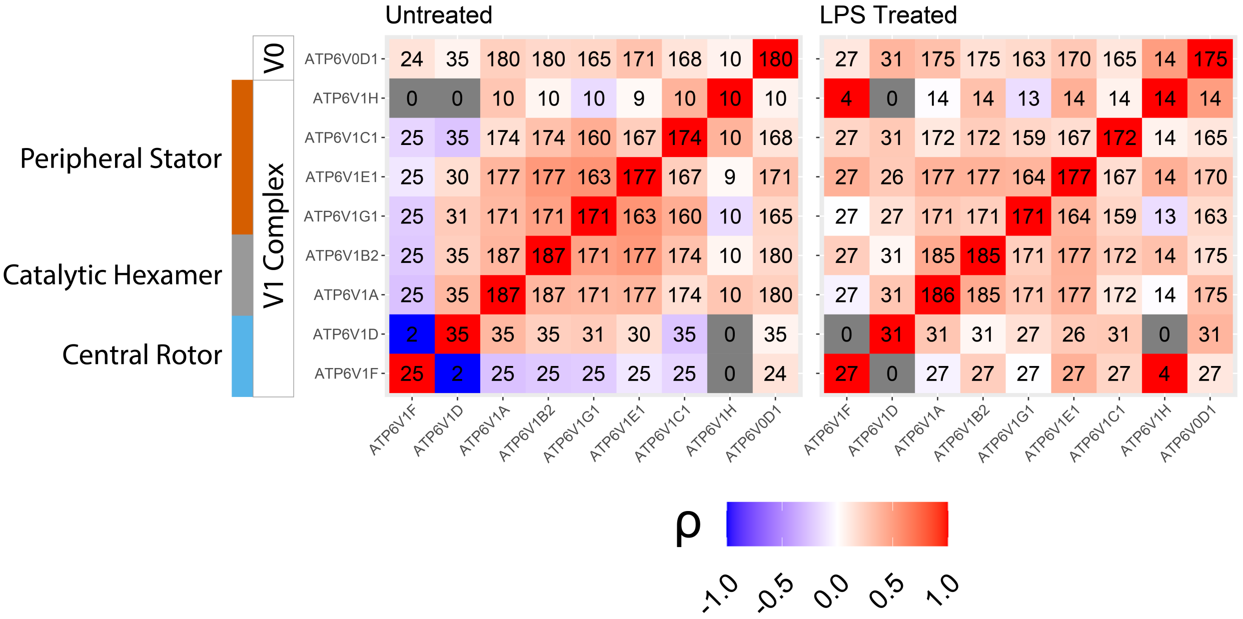

Supplemental Fig. S3 | Protein-protein correlations associated with the V-type ATPase Spearman correlations calculated for all quantified proteins associated with the V-type ATPase, two of which were excerpted in Fig. 4d. For each protein-protein correlation, the number of single cells in which both proteins were quantified is indicated within its respective heatmap tile. Structural annotations of these ATPase-associated proteins are indicated to the left of the untreated protein-protein heatmap.

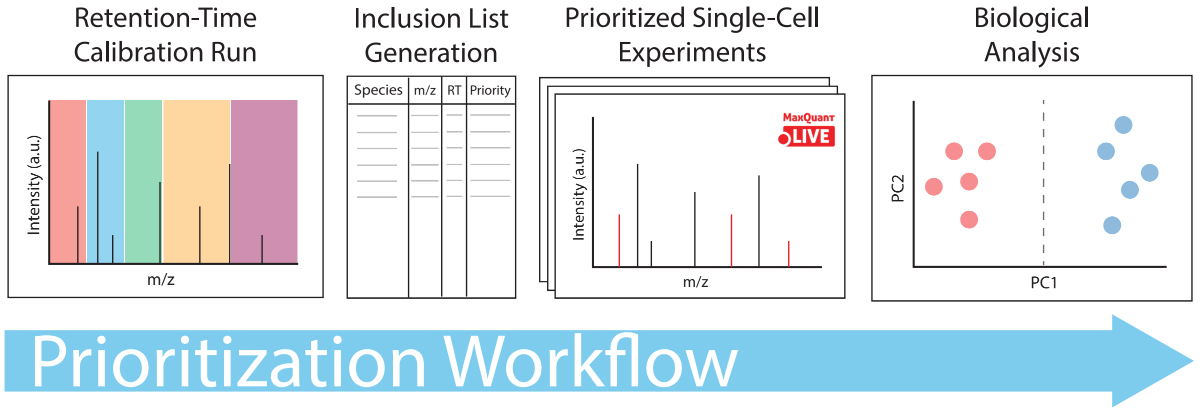

Supplemental Fig. S4 | pSCoPE workflow schematic

The pSCoPE workflow begins with the assembly of a number of precursors of experimental interest which can originate from prior DDA or DIA analyses of bulk samples, DDA analyses of previous SCoPE samples, or literature. A DIA method is then applied to a 1x injection of carrier and reference material to generate accurate retention times for the peptides of interest. The user then stratifies the peptides identified during the retention-time calibration run into priority levels, with the top priority level containing peptides of highest experimental interest. The prioritized inclusion list is imported into MaxQuant.Live for prioritized analysis of SCoPE samples.

Raw.file	AnnotationLayer	RI5	RI6	RI7	RI8	RI9	RI10	RI11	RI12	RI13	RI14	RI15	RI16	RI17	RI18
wGH0760	CellType	H	H	H	H	H	H	H	neg	M	M	M	M	M	M
	SpikeInPeptides	4x	4x	4x	2x	2x	2x	1x	1x	16x	16x	16x	8x	8x	8x
wGH0763	CellType	M	M	M	M	M	M	M	H	H	H	H	H	H	neg
	SpikeInPeptides	16x	16x	16x	8x	8x	8x	4x	4x	4x	2x	2x	2x	1x	1x
wGH0766	CellType	M	M	M	M	H	H	H	H	H	H	neg	M	M	M
	SpikeInPeptides	8x	8x	8x	4x	4x	4x	2x	2x	2x	1x	1x	16x	16x	16x
wGH0769	CellType	H	H	H	H	H	H	H	neg	M	M	M	M	M	M
	SpikeInPeptides	4x	4x	4x	2x	2x	2x	1x	1x	16x	16x	16x	8x	8x	8x
wGH0772	CellType	M	M	M	M	M	M	M	H	H	H	H	H	H	neg
	SpikeInPeptides	16x	16x	16x	8x	8x	8x	4x	4x	4x	2x	2x	2x	1x	1x
wGH0775	CellType	M	M	M	M	H	H	H	H	H	H	neg	M	M	M
	SpikeInPeptides	8x	8x	8x	4x	4x	4x	2x	2x	2x	1x	1x	16x	16x	16x
wGH0778	CellType	H	H	H	H	H	H	H	neg	M	M	M	M	M	M
	SpikeInPeptides	4x	4x	4x	2x	2x	2x	1x	1x	16x	16x	16x	8x	8x	8x
wGH0781	CellType	M	M	M	M	M	M	M	H	H	H	H	H	H	neg
	SpikeInPeptides	16x	16x	16x	8x	8x	8x	4x	4x	4x	2x	2x	2x	1x	1x

Supplemental Table S1 | Experimental design for spike-in analyses.
Schematic for the design of the single-cell sets used to benchmark reporter-ion quantitation in Fig. 3. Each row corresponds to a pSCoPE experiment with carriage-return-separated entries indicating cell type and spike-in concentration for each sample. The sample column headers denote the TMTpro 18plex label associated with each sample, with RI5 indicating 128C, RI6 indicating 129N, and so on. In the "cell type" section for each experiment, "H" denotes HEK293 cells, "M" denotes melanoma cells, and "neg" denotes negative control samples, which were identically processed to the single-cell samples, but which did not contain a cell.

Set	RI6	RI7	RI8	RI9	RI10	RI11	RI12	RI13	RI14	RI15	RI16
wGH0456	LPS	LPS	LPS	LPS	LPS	untreated	untreated	untreated	untreated	untreated	neg
wGH0457	untreated	untreated	untreated	untreated	neg	LPS	LPS	LPS	LPS	LPS	untreated
wGH0458	untreated	untreated	untreated	neg	LPS	LPS	LPS	LPS	untreated	LPS	untreated
wGH0459	LPS	LPS	LPS	untreated	untreated	untreated	untreated	untreated	LPS	neg	LPS
wGH0460	untreated	untreated	untreated	untreated	untreated	neg	LPS	LPS	LPS	LPS	LPS
wGH0461	untreated	untreated	untreated	untreated	neg	LPS	LPS	LPS	LPS	LPS	untreated
wGH0462	LPS	LPS	untreated	untreated	untreated	untreated	untreated	neg	LPS	LPS	LPS
wGH0463	untreated	untreated	untreated	neg	LPS	LPS	LPS	LPS	untreated	LPS	untreated
wGH0464	untreated	untreated	neg	LPS	LPS	LPS	LPS	LPS	untreated	untreated	untreated
wGH0465	LPS	untreated	untreated	untreated	untreated	untreated	untreated	LPS	LPS	LPS	LPS
wGH0466	LPS	LPS	untreated	untreated	untreated	untreated	untreated	neg	LPS	LPS	LPS
wGH0467	LPS	LPS	LPS	LPS	untreated	untreated	untreated	untreated	neg	untreated	LPS
wGH0468	untreated	untreated	untreated	neg	LPS	LPS	LPS	LPS	untreated	LPS	untreated
wGH0469	LPS	LPS	LPS	untreated	untreated	untreated	untreated	untreated	LPS	neg	LPS
wGH0470	untreated	untreated	untreated	untreated	neg	LPS	LPS	LPS	LPS	LPS	untreated
wGH0471	untreated	untreated	untreated	neg	LPS	LPS	LPS	LPS	untreated	LPS	untreated
wGH0472	LPS	LPS	LPS	LPS	LPS	untreated	untreated	untreated	untreated	untreated	neg
wGH0473	LPS	untreated	untreated	untreated	untreated	untreated	untreated	LPS	LPS	LPS	LPS
wGH0474	LPS	LPS	LPS	LPS	LPS	untreated	untreated	untreated	untreated	untreated	neg
wGH0475	untreated	untreated	untreated	untreated	neg	LPS	LPS	LPS	LPS	LPS	untreated
wGH0500	LPS	LPS	untreated	untreated	untreated	untreated	untreated	neg	LPS	LPS	LPS
wGH0501	LPS	LPS	LPS	LPS	untreated	untreated	untreated	untreated	neg	untreated	LPS
wGH0502	LPS	untreated	untreated	untreated	untreated	untreated	untreated	LPS	LPS	LPS	LPS
wGH0503	LPS	LPS	LPS	LPS	untreated	untreated	untreated	untreated	neg	untreated	LPS
wGH0504	LPS	untreated	untreated	untreated	untreated	untreated	untreated	LPS	LPS	LPS	LPS
wGH0505	untreated	untreated	untreated	neg	LPS	LPS	LPS	LPS	untreated	LPS	untreated
wGH0506	untreated	untreated	untreated	untreated	neg	LPS	LPS	LPS	LPS	LPS	untreated
wGH0507	LPS	LPS	LPS	LPS	untreated	untreated	untreated	untreated	neg	untreated	LPS
wGH0508	LPS	LPS	LPS	untreated	untreated	untreated	untreated	untreated	LPS	neg	LPS
wGH0509	untreated	untreated	untreated	untreated	neg	LPS	LPS	LPS	LPS	LPS	untreated
wGH0510	LPS	untreated	untreated	untreated	untreated	untreated	untreated	LPS	LPS	LPS	LPS
wGH0511	LPS	LPS	LPS	LPS	untreated	untreated	untreated	untreated	neg	untreated	LPS
wGH0512	LPS	untreated	untreated	untreated	untreated	untreated	untreated	LPS	LPS	LPS	LPS
wGH0513	LPS	LPS	LPS	untreated	untreated	untreated	untreated	untreated	LPS	neg	LPS
wGH0514	untreated	untreated	untreated	untreated	neg	LPS	LPS	LPS	LPS	LPS	untreated
wGH0515	LPS	LPS	LPS	LPS	untreated	untreated	untreated	untreated	neg	untreated	LPS
wGH0516	LPS	LPS	LPS	LPS	LPS	untreated	untreated	untreated	untreated	untreated	neg
wGH0517	LPS	LPS	untreated	untreated	untreated	untreated	untreated	neg	LPS	LPS	LPS
wGH0518	LPS	LPS	LPS	untreated	untreated	untreated	untreated	untreated	LPS	neg	LPS
wGH0519	untreated	untreated	neg	LPS	LPS	LPS	LPS	LPS	untreated	untreated	untreated
wGH0520	LPS	LPS	LPS	LPS	LPS	untreated	untreated	untreated	untreated	untreated	neg
wGH0521	LPS	untreated	untreated	untreated	untreated	untreated	untreated	LPS	LPS	LPS	LPS
wGH0522	LPS	LPS	untreated	untreated	untreated	untreated	untreated	neg	LPS	LPS	LPS
wGH0523	untreated	untreated	untreated	untreated	neg	LPS	LPS	LPS	LPS	LPS	untreated
wGH0524	LPS	LPS	LPS	LPS	untreated	untreated	untreated	untreated	neg	untreated	LPS
wGH0525	LPS	LPS	LPS	LPS	LPS	untreated	untreated	untreated	untreated	untreated	neg
wGH0526	LPS	LPS	LPS	LPS	LPS	untreated	untreated	untreated	untreated	untreated	neg
wGH0527	untreated	untreated	untreated	untreated	untreated	neg	LPS	LPS	LPS	LPS	LPS
wGH0528	untreated	untreated	untreated	untreated	untreated	neg	LPS	LPS	LPS	LPS	LPS
wGH0529	untreated	untreated	neg	LPS	LPS	LPS	LPS	LPS	untreated	untreated	untreated
wGH0530	LPS	LPS	untreated	untreated	untreated	untreated	untreated	neg	LPS	LPS	LPS
wGH0531	untreated	untreated	untreated	neg	LPS	LPS	LPS	LPS	untreated	LPS	untreated
wGH0532	untreated	untreated	neg	LPS	LPS	LPS	LPS	LPS	untreated	untreated	untreated
wGH0533	untreated	untreated	untreated	untreated	untreated	neg	LPS	LPS	LPS	LPS	LPS
wGH0534	untreated	untreated	untreated	neg	LPS	LPS	LPS	LPS	untreated	LPS	untreated
wGH0535	untreated	untreated	neg	LPS	LPS	LPS	LPS	LPS	untreated	untreated	untreated
wGH0536	LPS	LPS	untreated	untreated	untreated	untreated	untreated	neg	LPS	LPS	LPS
wGH0537	LPS	LPS	LPS	LPS	untreated	untreated	untreated	untreated	neg	untreated	LPS
wGH0538	LPS	LPS	LPS	untreated	untreated	untreated	untreated	untreated	LPS	neg	LPS
wGH0539	LPS	untreated	untreated	untreated	untreated	untreated	untreated	LPS	LPS	LPS	LPS

Supplemental Table S2 | Experimental design for single-cell BMDM analyses.
Schematic for the design of the single-cell sets composing the bone-marrow-derived macrophage (BMDM) analyses associated with Figs. 4/5/6. Each row corresponds to a pSCoPE experiment. The sample column headers denote the TMTpro 16plex label associated with each sample, with RI6 indicating 129N, RI7 indicating 129C, and so on. “LPS” denotes BMDMs that were stimulated with LPS for 24 hours, “untreated” denotes BMDMs that were not stimulated with LPS, and “neg” denotes negative control samples, which were identically processed to the single-cell samples, but which did not contain a cell. RI1 contained the carrier sample; RI2 contained the reference sample; RI3 and 4 were unused due to isotopic contamination from the carrier and reference channels, respectively; single-cell samples labeled with RI5 were unused in downstream analysis due to isotopic contamination from the carrier channel evident during QC steps.

MS1 Settings:	Method 1	Method 2	Method 3	Method 4	Method 5
Polarity:	Positive	Positive	Positive	Positive	Positive
Resolution:	140k	140k	140k	70k	140k
AGC Target:	3e6	3e6	3e6	3e6	3e6
Max Injection Time:	512ms	512ms	512ms	300ms	512ms
Scan Range (Th):	450-1600	450-1600	450-1258	478-1470	400-800
DIA Scan Settings:					
Default Charge State	2	2	2	2	2
Polarity	Positive	Positive	Positive	Positive	Positive
Resolution	35k	35k	35k	35k	17.5k
AGC target	5e5	5e5	5e5	3e6	5e5
Max Injection Time	auto	auto	auto	110ms	64ms
MSX Count	1	1	1	1	1
MSX isochronous	on	on	on	on	on
Fixed first mass	200	200	200	200	200
NCE	33	33	33	27	27
DIA window scheme	1	2	3	4	5

Supplemental Table S3 | MS Methods for DIA Acquisitions: Parameters for data acquisition for the DIA methods used in this publication. Accompanying DIA window schemes can be found in Supporting Table S5. Chromatographic methods used with these DIA methods can be found in Supporting Table S4. The text in black denotes parameters which are in common with the default method (Method 1), while the red text denotes parameters which vary from the default method.

Time:	Meth. 1	Time	Meth. 2	Time	Meth. 3	Time	Meth. 4
0 min.	4% B	0 min.	4% B	0 min.	4% B	0 min.	4% B
4 min.	4% B	4 min.	4% B	4 min.	4% B	4 min.	4% B
12 min.	8% B	12 min.	8% B	12 min.	7% B	22 min.	8% B
75 min.	35% B	75 min.	35% B	135 min.	32% B	130 min.	29% B
77 min.	95% B	77 min.	95% B	137 min.	95% B	134 min.	95% B
80.1 min.	4% B	80.1 min.	4% B	140.1 min.	4% B	140.1 min.	4% B
95 min.	4% B	105 min.	4% B	160 min.	4% B	155 min.	4% B
end	4% B	end	4% B	end	4% B	end	4% B

Supplemental Table S4 | Gradient Methods for DIA Acquisitions: Chromatographic parameters for the DIA methods used in this publication. Accompanying instrument parameters can be found in Supporting Table S3.

DIA Window Scheme	1	2	3	4	5
Window 1:					
Loop Count:	21	21	21	25	20
Isolation Window (Th):	20	15	20	12.5	19.5
Spans (Th):	450-860	450-755	450-860	450-778.5	400-799.5
Window 2:					
Loop Count:	7	8	8	7	
Isolation Window (Th):	50	20	50	25	
Spans (Th):	859.5-1206.5	754.5-911	859.5-1256	778 - 950	
Window 3:					
Loop Count:	1	6		8	
Isolation Window (Th):	394	50		62.5	
Spans (Th):	1206 - 1600	910.5-1208		949.5-1446	
Window 4:					
Loop Count:		1			
Isolation Window (Th):		393			
Spans (Th):		1207.5-1600.5			

Supplemental Table S5 | DIA window schemes for DIA acquisitions: This table contains DIA window parameters referenced in the associated instrument methods detailed in Supporting Table S3 for the DIA methods used in this publication.

MS1 settings:	Method 1	Method 2	Method 3	Method 4	Method 5
Resolution	70k	70k	70k	70k	70k
AGC Target	1e6	1e6	1e6	1e6	1e6
Injection Time	100ms	100ms	100ms	100ms	100ms
Scan Range (Th)	450-1600	450-1600	450-1600	450-1258	450-1258
**MS2 settings:**					
Resolution	70k	35k	70k	140k	140k
AGC Target	5e4	5e4	5e4	5e4	
Injection Time	300 ms	150 ms	300ms	500 ms	600 ms
TopN	7	14	7	4	4
Isolation Window (Th)	0.7	0.7	0.5	0.5	0.5
Offset (Th)	0.3	0.3	0	0	0
Fixed First Mass (Th)	100	100	100	100	100
NCE	33	33	33	33	33
Spectrum Type	Centroid	Centroid	Centroid	Centroid	Centroid
Min. AGC Target	2e4	2e4	2e4	2e4	2e4
Apex Triggering	FALSE	FALSE	FALSE	FALSE	FALSE
Charge Exclusion	Unknown, 1, and > 6	Unknown, 1, and > 6	Unknown, 1, and > 6	Unknown, 1, and > 6	Unknown, 1, and > 6
Peptide Match	FALSE	FALSE	FALSE	FALSE	FALSE
Exclude Isotopes	Enabled	Enabled	Enabled	Enabled	Enabled
Dynamic Exclusion (sec)	30	30	30	30	30

Supplemental Table S6 | MS methods for Shotgun Acquisitions: Parameters for data acquisition for the shotgun methods used in this publication. Chromatographic methods used with these shotgun methods can be found in Supporting Table S7. The text in black denotes parameters which are in common with the default method (Method 1), while the red text denotes parameters which vary from the default method.

Time:	Methods 1	Time	Method 2
0 min.	4% B	0 min.	4% B
4 min.	4% B	4 min.	4% B
12 min.	8% B	12 min.	8% B
75 min.	35% B	75 min.	35% B
77 min.	95% B	77 min.	95% B
80.1 min.	4% B	80.1 min.	4% B
95 min.	4% B	105 min.	4% B
end	4% B	end	4% B

Supplemental Table S7 | Gradient Methods for Shotgun Acquisitions: Chromatographic parameters for the shotgun methods used in this publication. Accompanying instrument parameters can be found in Supporting Table S6.

Purpose	Sample	MS Method	Gradient	Inclusion List	Figure
Spectral Library	SQC, 10x	DIA:1	1		
Spectral Library	SQC, 10x	DIA:2	1		
Spectral Library	SQC, 1x	DIA:1	1		
Spectral Library	SQC, 1x	DIA:1	1		
RT Calibration	SQC, 1x	DIA:1	1		
Scout	SQC, 1x	pSCoPE:1	1	1	
Scout	SQC, 1x	pSCoPE:1	1	2	
Scout	SQC, 1x	pSCoPE:1	1	3	
Scout	SQC, 1x	pSCoPE:1	1	4	
pSCoPE Benchmark	SQC, 1x	MaxQuant.Live:1	1	5	1
pSCoPE Benchmark	SQC, 1x	pSCoPE:1	1	5	1

Supplemental Table S8 | Method and Sample Mapping: MaxQuant.Live Contrast Experiments, Fig. 1: This table connects the experiments performed as part of Figure 1 with information regarding their sample type, instrument method, gradient, and inclusion list. More details regarding the instrument methods (Supporting Table S3, Supporting Table S6, Supporting Table S11), gradients (Supporting Table S4 and Supporting Table S7), and inclusion lists can be found in their respective tables and sections.

Purpose	Sample	MS Method	Gradient	Inclusion List	Figure
Spectral Library	Carrier and Ref., 10x	DIA:1	1		
Spectral Library	Carrier and Ref., 10x	DIA:2	1		
Spectral Library	Carrier and Ref., 1x	DIA:1	1		
Spectral Library	Carrier and Ref., 1x	DIA:1	1		
RT Calibration	Carrier and Ref., 1x	DIA:1	1		
Scout	Carrier and Ref., 1x	pSCoPE:2	1	6	
Scout	Carrier and Ref., 1x	pSCoPE:2	1	7	
Scout	Carrier and Ref., 1x	pSCoPE:2	1	8	
Scout	Carrier and Ref., 1x	pSCoPE:2	1	9	
pSCoPE Benchmark	Single cell (nPOP)	Shotgun:3	1		2
pSCoPE Benchmark	Single cell (nPOP)	pSCoPE:2	1	10 and 11	2
Spike-in Analysis	Single cell (nPOP)	pSCoPE:3	1	12	3

Supplemental Table S9 | Method and Sample Mapping: HEK and Melanoma Experiments, Figs. 2/3: This table connects the experiments performed as part of Figures 2 and 3 with information regarding their sample type, instrument method, gradient, and inclusion list. More details regarding the instrument methods (Supporting Table S3, Supporting Table S6, and Supporting Table S12), gradients (Supporting Table S4 and Supporting Table S7), and inclusion lists can be found in their respective tables and sections.

Purpose	Sample	MS Method	Gradient	Inclusion List	Figure
Differential Protein Analysis	1,800 cells from each condition (LPS and control)	DDA:1	1		
Differential Protein Analysis	1,800 cells from each condition (LPS and control)	DDA:2	1		
Differential Protein Analysis	1,000 cells from each condition (LPS and control), separately	DIA:1	1		
Endocytosis Assay	10,000 cells from each condition (LPS and control), separately	DIA:5	4		5b
MEROPS Validation	1μg from each condition (LPS and control), separately	DIA:4	3		6a
Spectral Library	Carrier and Ref., 5x	DIA:3	1		
Spectral Library	Carrier and Ref., 1x	DIA:3	1		
RT Calibration	Carrier and Ref., 1x	DIA:3	1		
Scout	Carrier and Ref., 1x	pSCoPE:4	1	13	
pSCoPE Benchmark	Single cell (nPOP)	Shotgun:4	1		
pSCoPE Benchmark	Single cell (nPOP)	pSCoPE:5	1	14	4,5,6
Method Development	Single cell (mPOP)	Shotgun:5	2		
Method Development	Single cell (mPOP)	pSCoPE:6	2	15	

Supplemental Table S10 | Method and Sample Mapping: BMDM samples, Figs. 4/5/6: This table connects the experiments performed as part of Figures 4/5/6 with information regarding their sample type, instrument method, gradient, and inclusion list. More details regarding the instrument methods (Supporting Table S3, Supporting Table S6, Supporting Table S13, and Supporting Table S14), gradients (Supporting Table S4 and Supporting Table S7), and inclusion lists can be found in their respective tables and sections.

Global settings: Survey scan	Scout Runs and Fig. 1 (Method 1)
ScanDataAsProfile	True
PositiveMode	True
MaxIT	100ms
Resolution	70,000
AgcTarget	1,000,000
MzRange	(450,1600)
BoxCarScans	0
**Global settings: TopN**	
NumOfMS2Scans	0
**RealtimeCorrection**	**Scout Runs and Fig. 1 (Method 1)**
MzTolerances	(4.5,5)
RetentionTimeTolerances	(0.01,2)
SigmaScaleFactorRt	3
PeptideHistoryLength	2
MinUsedCorrectionPeptides	50
IntensityPeakRatioThreshold	1e-5
PeptideDetectionIsoPeaks	2
IsotopeTolerance	9
Ms2DetectionNeeded	False
Ms2ExcludeDetectedPeptides	False
Ms2MinNormIntensity	0.1
Ms2MzTolerance	20
PIFWindowSize	1.4
**TargetedMs2**	**Scout Runs and Fig. 1 (Method 1)**
BatMode	False
AutoPriority	True
DefaultPriority	0
MaxNumOfScans	1
WindowAndOffsetInDalton	False
ScanDataAsProfile	False
WindowSize	0.5
MzOffset	0
LowerMzBound	100
CollisionEnergy	33
LifeTime	2,400ms
Resolution	70,000
MaxIT	300ms
AgcTarget	3,000,000
PositiveMode	True

Supplemental Table S11 | Prioritized acquisition parameters for benchmarking MaxQuant.Live with and without prioritization, Fig. 1 This table contains the MaxQuant.Live parameters used in experiments associated with Figure 1. The experiment-type-to-method mapping for these methods can be found in the corresponding table (Supporting Table S8).

Global settings: Survey scan	Scout Runs and Fig. 2 (Method 2)	Fig. 3 (Method 3)
ScanDataAsProfile	True	True
PositiveMode	True	True
MaxIT	100ms	100ms
Resolution	70,000	70,000
AgcTarget	1,000,000	1,000,000
MzRange	(450,1600)	(450,1600)
BoxCarScans	0	0
**Global settings: TopN**		
NumOfMS2Scans	0	0
**RealtimeCorrection**	**Scout Runs and Fig. 2 (Method 2)**	**Fig. 3 (Method 3)**
MzTolerances	(4.5,5)	(4.5,5)
RetentionTimeTolerances	(0.01,2)	(0.01,2)
SigmaScaleFactorRt	3	3
PeptideHistoryLength	2	2
MinUsedCorrectionPeptides	50	50
IntensityPeakRatioThreshold	1e-5	1e-5
PeptideDetectionIsoPeaks	2	2
IsotopeTolerance	9	9
Ms2DetectionNeeded	False	False
Ms2ExcludeDetectedPeptides	False	False
Ms2MinNormIntensity	0.1	0.1
Ms2MzTolerance	20	20
PIFWindowSize	1.4	1.4
**TargetedMs2**	**Scout Runs and Fig. 2 (Method 2)**	**Fig. 3 (Method 3)**
BatMode	False	False
AutoPriority	True	True
DefaultPriority	0	0
MaxNumOfScans	1	1
WindowAndOffsetInDalton	False	False
ScanDataAsProfile	False	False
WindowSize	0.5	0.5
MzOffset	0	0
LowerMzBound	100	100
CollisionEnergy	33	33
LifeTime	2,100ms	2,400ms
Resolution	70,000	70,000
MaxIT	300ms	300ms
AgcTarget	3,000,000	3,000,000
PositiveMode	True	True

Supplemental Table S12 | Prioritized acquisition parameters for HEK and melanoma single-cell sets, Fig. 2/3: This table contains the MaxQuant.Live parameters used in experiments associated with Figures 2/3. The experiment-type-to-method mapping for these methods can be found in the corresponding table (Supporting Table S9).

Global settings: Survey scan	Scout sample (Method 4)	Single-cell sets (Method 5)
ScanDataAsProfile	True	True
PositiveMode	True	True
MaxIT	100ms	100ms
Resolution	70,000	70,000
AgcTarget	1,000,000	1,000,000
MzRange	(450,1258)	(450,1258)
BoxCarScans	0	0
**Global settings: TopN**	**Scout sample (Method 4)**	**Single-cell sets (Method 5)**
NumOfMS2Scans	0	0
**RealtimeCorrection**	**Scout sample (Method 4)**	**Single-cell sets (Method 5)**
MzTolerances	(4.5,5)	(4.5,5)
RetentionTimeTolerances	(0.01,2)	(0.01,2)
SigmaScaleFactorRt	3	3
PeptideHistoryLength	2	2
MinUsedCorrectionPeptides	15	15
IntensityPeakRatioThreshold	1e-5	1e-5
PeptideDetectionIsoPeaks	2	2
IsotopeTolerance	9	9
Ms2DetectionNeeded	False	False
Ms2ExcludeDetectedPeptides	False	False
Ms2MinNormIntensity	0.1	01
Ms2MzTolerance	20	20
**TargetedMs2**	**Scout sample (Method 4)**	**Single-cell sets (Method 5)**
BatMode	False	False
AutoPriority	True	True
DefaultPriority	0	0
MaxNumOfScans	1	1
WindowAndOffsetInDalton	False	False
ScanDataAsProfile	False	False
WindowSize	0.5	0.5
MzOffset	0	0
LowerMzBound	100	100
CollisionEnergy	33	33
LifeTime	2,400ms	2,100ms
Resolution	140,000	140,000
MaxIT	500ms	500ms
AgcTarget	1,000,000	1,000,000
PositiveMode	True	True

Supplemental Table S13 | Prioritized acquisition parameters for nPOP BMDM single-cell samples, Fig. 4/5/6: This table contains the MaxQuant.Live parameters used in experiments associated with Figures 4/5/6. The experiment-type-to-method mapping for these methods can be found in the corresponding table (Supporting Table S10).

Global settings: Survey scan	mPOP Single-cell sets (Method 6)
ScanDataAsProfile	True
PositiveMode	True
MaxIT	100ms
Resolution	70,000
AgcTarget	1,000,000
MzRange	(450,1258)
BoxCarScans	0
**Global settings: TopN**	**mPOP Single-cell sets (Method 6)**
NumOfMS2Scans	0
**RealtimeCorrection**	**mPOP Single-cell sets (Method 6)**
MzTolerances	(4.5,5)
RetentionTimeTolerances	(0.01,1.5)
SigmaScaleFactorRt	3
PeptideHistoryLength	2
MinUsedCorrectionPeptides	15
IntensityPeakRatioThreshold	1e-5
PeptideDetectionIsoPeaks	2
IsotopeTolerance	9
Ms2DetectionNeeded	False
Ms2ExcludeDetectedPeptides	False
Ms2MinNormIntensity	0.1
Ms2MzTolerance	20
**TargetedMs2**	**mPOP Single-cell sets (Method 6)**
BatMode	False
AutoPriority	True
DefaultPriority	0
MaxNumOfScans	1
WindowAndOffsetInDalton	False
ScanDataAsProfile	NA
WindowSize	0.5
MzOffset	0
LowerMzBound	100
CollisionEnergy	33
LifeTime	2,400ms
Resolution	140,000
MaxIT	600ms
AgcTarget	1,000,000
PositiveMode	True

Supplemental Table S14 | Prioritized acquisition parameters for mPOP BMDM single-cell samples: This table contains the MaxQuant.Live parameters used in preliminary experiments. The experiment-type-to-method mapping for these methods can be found in the corresponding table (Supporting Table S10).
