# Supplementary material for: Prioritized mass spectrometry increases the depth, sensitivity and data completeness of single-cell proteomics
Source: Nat Methods. 2023 Apr 3;20(5):714–22. doi: 10.1038/s41592-023-01830-1 (PMC10172113; doi:10.1038/s41592-023-01830-1)
Supplement: Supplementary file 1 [file 41592_2023_1830_MOESM2_ESM.pdf]

nature portfolio

Corresponding author(s): Nikolai Slavov

Last updated by author(s): Jan 5, 2023

Reporting Summary

Nature Portfolio wishes to improve the reproducibility of the work that we publish. This form provides structure for consistency and transparency in reporting. For further information on Nature Portfolio policies, see our Editorial Policies and the Editorial Policy Checklist.

Statistics

For all statistical analyses, confirm that the following items are present in the figure legend, table legend, main text, or Methods section.

n/a	Confirmed	
☐	☒	The exact sample size (*n*) for each experimental group/condition, given as a discrete number and unit of measurement
☐	☒	A statement on whether measurements were taken from distinct samples or whether the same sample was measured repeatedly
☐	☒	The statistical test(s) used AND whether they are one- or two-sided<br>*Only common tests should be described solely by name; describe more complex techniques in the Methods section.*
☐	☒	A description of all covariates tested
☐	☒	A description of any assumptions or corrections, such as tests of normality and adjustment for multiple comparisons
☐	☒	A full description of the statistical parameters including central tendency (e.g. means) or other basic estimates (e.g. regression coefficient) AND variation (e.g. standard deviation) or associated estimates of uncertainty (e.g. confidence intervals)
☐	☒	For null hypothesis testing, the test statistic (e.g. *F*, *t*, *r*) with confidence intervals, effect sizes, degrees of freedom and *P* value noted<br>*Give P values as exact values whenever suitable.*
☒	☐	For Bayesian analysis, information on the choice of priors and Markov chain Monte Carlo settings
☐	☒	For hierarchical and complex designs, identification of the appropriate level for tests and full reporting of outcomes
☐	☒	Estimates of effect sizes (e.g. Cohen's *d*, Pearson's *r*), indicating how they were calculated

Our web collection on statistics for biologists contains articles on many of the points above.

Software and code

Policy information about availability of computer code

Data collection	All mass-spec data were collected by Xcalibur™ (V_4.2, V_4.3), which is commercial software by Thermo Fisher Scientific, or MaxQuant.live (version 2.1), a published software platform for data acquisition on Thermo Quadrupole instruments (DOI:https://doi.org/10.1074/mcp.TIR118.001131)
Data analysis	Raw DDA mass-spec data were searched by MaxQuant (V_2.0.30, V_1.6.7.0, V_1.6.17.0) and all DIA data were searched by Spectronaut (v_15.1, v_15.0, v_14.1, v_14.10 & v_15.6) or DIA-NN (V_1.8.2 beta 2, V_1.8.1 beta 23)<br>The output of MaxQuant, Spectronaut, and DIA-NN were further analyzed by a data processing pipeline implemented in the R programming language, which is available at https://github.com/SlavovLab/pSCoPE.

For manuscripts utilizing custom algorithms or software that are central to the research but not yet described in published literature, software must be made available to editors and reviewers. We strongly encourage code deposition in a community repository (e.g. GitHub). See the Nature Portfolio guidelines for submitting code & software for further information.

Data

Policy information about availability of data

All manuscripts must include a data availability statement. This statement should provide the following information, where applicable:

- Accession codes, unique identifiers, or web links for publicly available datasets
- A description of any restrictions on data availability
- For clinical datasets or third party data, please ensure that the statement adheres to our policy

MassIVE MSV000090383 (https://massive.ucsd.edu/ProteoSAFe/dataset.jsp?task=76188b5c13714a6da059d2845fca1c64)

Field-specific reporting

Please select the one below that is the best fit for your research. If you are not sure, read the appropriate sections before making your selection.

☒ Life sciences ☐ Behavioural & social sciences ☐ Ecological, evolutionary & environmental sciences

For a reference copy of the document with all sections, see nature.com/documents/nr-reporting-summary-flat.pdf

Life sciences study design

All studies must disclose on these points even when the disclosure is negative.

Sample size

For the benchmarking experiments associated with Figure 1b/e:
1 sample of TMT-labeled serially diluted digest featuring 6 single-cell-level samples (3 U937; 3 HEK293) with 12 replicate injections

For the benchmarking experiments associated with Figure 2abcde:
97 cells associated with the pSCoPE sets shown in Figure 2a (50 HEK293 cells; 47 melanoma cells)
103 cells associated with the pSCoPE sets shown in Figure 2b/c/d/e (51 HEK293 cells; 52 melanoma cells)
97 cells associated with the shotgun sets shown in Figure 2abcd (46 HEK293 cells; 51 melanoma cells)

For the spike-in analysis associated with Figure 3:
112 samples associated with the pSCoPE sets shown in Figure 2b (51 HEK293 cells; 53 melanoma cells; 8 control samples)

For the BMDM samples associated with Figures 4/5/6:
373 cells associated with the pSCoPE sets (187 untreated BMDMs; 186 24-hr LPS-treated BMDMs)

Sample sizes were chosen to include at least a dozen single cells for technical experiment to support the validity of the results and a couple hundred single cells from the primary macrophages to give us more statistical power to identify biological differences and regulatory mechanisms.

Data exclusions

Single cells were excluded from downstream analysis if their quantification variability (the coefficient of variation of relative peptide abundances which mapped to the same protein) exceeded 0.4, except in the case of the spike-in experiments, where the reporter ion intensities derived from the spike-in peptides were of primary interest.

Replication

For the benchmarking experiments associated with Figure 1b/c:
6 MaxQuant.Live experiments without prioritization enabled
6 MaxQuant.Live experiments with prioritization enabled

For the benchmarking experiments associated with Figure 2abcde:
8 pSCoPE experiments shown in Figure 2a
8 pSCoPE experiments shown in Figure 2b/c/d/e
8 Shotgun experiments shown in Figure 2a/b/c/d

For the spike-in analysis associated with Figure 3:
8 pSCoPE experiments

For the BMDM samples associated with Figures 4/5/6:
40 pSCoPE experiments

*Each experiment contained up to 14 single-cell samples

Randomization

Single cells, control samples, and TMT-label application were randomized within each 14 drop array that constituted an experimental set for the HEK293 and Melanoma samples shown in Figure 2 and the BMDM samples shown in Figures 4/5/6, prepared by nano-ProteOmic sample Preparation (nPOP). For the HEK293 and melanoma single-cell samples and control samples shown in Figure 3, the spike-in peptide concentration was also randomized across samples.

Blinding

Blinding is not relevant to this study because having prior knowledge of which samples are being tested cannot change the raw data or the down-stream results.

Reporting for specific materials, systems and methods

We require information from authors about some types of materials, experimental systems and methods used in many studies. Here, indicate whether each material, system or method listed is relevant to your study. If you are not sure if a list item applies to your research, read the appropriate section before selecting a response.

Materials & experimental systems

n/a	Involved in the study
☒	☐ Antibodies
☐	☒ Eukaryotic cell lines
☒	☐ Palaeontology and archaeology
☐	☒ Animals and other organisms
☒	☐ Human research participants
☒	☐ Clinical data
☒	☐ Dual use research of concern

Methods

n/a	Involved in the study
☒	☐ ChIP-seq
☐	☒ Flow cytometry
☒	☐ MRI-based neuroimaging

Eukaryotic cell lines

Policy information about cell lines

Cell line source(s)	HEK293 cell lines purchased from ATCC (CRL-1573); melanoma cells (WM989-A6-G3) were a gift from Sydney Shaffer, University of Pennsylvania
Authentication	ATCC authentication report based on PCR assays with species-specific primers
Mycoplasma contamination	The cells were not tested for mycoplasma contamination
Commonly misidentified lines (See ICLAC register)	None

Animals and other organisms

Policy information about studies involving animals; ARRIVE guidelines recommended for reporting animal research

Laboratory animals	C57BL/6J (Jax 000664) mice (female, 8 weeks old) were purchased from The Jackson Laboratory and used to generate the bone-marrow-derived macrophages
Wild animals	Please state here that no wild animals were used in the study.
Field-collected samples	Please state here that no field collected samples were used in the study.
Ethics oversight	All protocols were approved by the Institutional Animal Care and Use Committee (IACUC) of Boston Children's Hospital.

Note that full information on the approval of the study protocol must also be provided in the manuscript.

Flow Cytometry

Plots

Confirm that:

☒ The axis labels state the marker and fluorochrome used (e.g. CD4-FITC).

☒ The axis scales are clearly visible. Include numbers along axes only for bottom left plot of group (a 'group' is an analysis of identical markers).

☒ All plots are contour plots with outliers or pseudocolor plots.

☒ A numerical value for number of cells or percentage (with statistics) is provided.

Methodology

Sample preparation	C57BL/6J (Jax 000664) mice were purchased from The Jackson Laboratory. Bone-marrow-derived macrophages (BMDMs) were differentiated from bone marrow in Dulbecco’s modified Eagle medium (DMEM; Thermo Fisher Scientific), 30% L929-M-CSF supernatant and 10% fetal bovine serum (FBS). After 7 days, BMDMs were replated at 1x10^6 cells/ml in DMEM supplemented with 10% FBS, and each plate was either stimulated for 24 hours with LPS (Serotype O55:B5, Enzo Life Sciences) at 1 ug/ml or allowed to rest.<br><br>Murine BMDMs were then incubated with dextran conjugated to Alexa Fluor 568 (Thermo, D22912) at a final concentration of 0.5 mg/ml for 45 minutes at 37 degrees C. After the incubation period, cells were washed twice with 1x PBS and incubated with PBS-2mM EDTA to detach from the plate. Prior to FACS analysis, cells were spun down at 300g for 5 minutes and washed with 1x PBS before being resuspended. Using a Sony MA900, Dextran-AF568 fluorescence was then analyzed for cells from each treatment condition, and a minimum of 70,000 cells from the top and bottom 10% of the FITC-AF568 fluorescence distribution were then sorted for downstream sample preparation and mass spectrometry analysis.
Instrument	Sony MA900

Software	Cell Sorter Software
Cell population abundance	~80,000 cells per dextran uptake condition (bottom dextran-AF568 decile and top dextran-AF568 decile) per treatment condition (untreated and treated with LPS for 24 hours)
Gating strategy	The following gating strategy was used to separate singlets from debris: FSC-A from 75k to 210k, SSC-A from 35k to 170k (visible in supplemental figure 7).<br><br>For the untreated BMDMs, cells from the bottom 10.3% and top 11.0% of dextran:AP568 fluorescence distribution were isolated for the low and high uptake subpopulations, respectively. For the 24-hr LPS-stimulated BMDMs, cells from the bottom 11.6% and the top 12.0% of dextran: AP568 fluorescence distribution were isolated for the low and high uptake subpopulations, respectively

☒ Tick this box to confirm that a figure exemplifying the gating strategy is provided in the Supplementary Information.
